# Add-on hemoperfusion in SARS-CoV-2-infected pregnant patients: a case series

**DOI:** 10.1186/s13256-024-04726-6

**Published:** 2024-09-03

**Authors:** Supattra Chiewroongroj, Thummaporn Naorungroj, Ranistha Ratanarat

**Affiliations:** 1 Department of Medicine, Faculty of Medicine Siriraj Hospital, Mahidol University, Bangkok, 10700 Thailand; 2 Division of Critical Care Medicine, Department of Medicine Siriraj Hospital, Mahidol University, 2, Wanglang Rd, Bangkoknoi, Bangkok, 10700 Thailand

**Keywords:** COVID-19, Pregnancy, Hemoperfusion, Immunomodulation, Case report

## Abstract

**Background:**

Pregnant women are more likely to have a higher severity of illness after being infected with coronavirus disease 2019 compared with the general population, particularly in the hyperinflammatory phase. However, immunomodulatory drugs are contraindicated and have been associated with an increased risk of fetal abnormalities. Therefore, we are reporting our experience with the use of HA330 hemoperfusion in combination with standard therapy in severe to critical coronavirus disease 2019 cases among pregnant patients.

**Case presentation:**

From January 2020 to December 2021, four pregnant Thai women were treated with hemoperfusion using a cytokine adsorptive technique. The patients’ ages ranged from 21 to 36 years old, and their gestational ages at the time of admission ranged from 18 to 38 weeks. Two patients required intubation. Extracorporeal blood purification with an adsorptive cartridge (HA330^®^, Jafron, China) was applied as an adjunctive strategy to standard therapy approximately one week after the onset of symptoms, and most patients received three sessions of hemoperfusion. The baseline C-reactive protein level was greater than 80 mg/dL. The results showed that hemoperfusion could decrease the C-reactive protein level by approximately 80% and improve oxygenation. The newborns were delivered by Cesarean section without complications. Neither mortality nor serious adverse events related to hemoperfusion occurred.

**Conclusion:**

This report may help ensure the use of the hemoperfusion strategy in pregnant patients during a cytokine storm. However, a larger cohort is needed to confirm its safety and efficacy.

## Introduction

Since December 2019, the emerging virus identified as severe acute respiratory syndrome coronavirus 2 (SARS-CoV-2) has spread worldwide and caused a global pandemic called coronavirus disease 2019 (COVID-19), with a mortality rate of up to 49% in critical cases [1]. Pregnant women are more susceptible to COVID-19 disease compared with the general population [2]. The physiological maternal immunological state undergoes active adaptation and modification during the different stages of pregnancy [3]. Gestation is characterized by increased estrogen and progesterone levels, leading to swelling of the upper respiratory tract, as well as restricted lung expansion due to a gravid uterus, which increases the risk of respiratory infections and compromise [4, 5].

The hyperinflammatory response, also known as cytokine storm, has been associated with the development and progression of acute respiratory distress syndrome (ARDS), septic shock, and multiorgan failure and is a significant cause of death in COVID-19 disease [6–8]. Suppressing these mediators using immunomodulators or removing them through extracorporeal blood purification is a hypothesis that could potentially reduce related complications. However, tocilizumab, an interleukin-6 receptor inhibitor, which is a standard adjunctive therapy in severe COVID-19 with high levels of C-reactive protein (CRP) [9], is relatively contraindicated in pregnancy. On the other hand, hemoperfusion (HP) with a cytokine adsorbent, aiming to remove inflammatory mediators, has shown benefits in the treatment of dysregulated inflammatory conditions [10]. Additionally, the US Food and Drug Administration (FDA) has granted emergency use authorization (EUA) for a blood purification system to treat patients with confirmed COVID-19 and respiratory failure [11]. Previous studies in patients with severe COVID-19 pneumonia have demonstrated that HP with a cytokine adsorber (HA330^®^, Jafron, China) significantly removes inflammatory factors (interleukin-6 [IL-6], CRP, and ferritin) from the circulation, decreases hospital mortality and length of stay, improves oxygenation, and poses no safety concerns [12–16]. However, these trials did not include pregnant patients.

In light of this, we report four cases of pregnant patients with severe COVID-19 to demonstrate the utilization of HP as an adjunctive therapy during the hyperinflammatory phase.

## Case presentation

### Patients

During the pandemic era (from January 2020 to December 2021), four pregnant Thai women were treated with HP using a cytokine adsorptive cartridge. All patients were admitted to the medical ICU and diagnosed with COVID-19 through polymerase chain reaction (PCR) testing, classified as severe to critical COVID-19 disease according to the WHO classification [17]. Only one case (patient number 2) had underlying disorders, including end-stage renal disease (ESRD) due to chronic glomerulonephritis (GN), a history of cadaveric donor kidney transplantation, chronic hepatitis B infection, hypertension, and hypoparathyroidism following parathyroidectomy. The patients’ baseline characteristics are summarized in Table 1.

**Table 1:** Baseline characteristics of patients in this study

	Patient 1		Patient 2		Patient 3		Patient 4	
Age, years	21		36		25		27	
GA at admission, weeks	18^+3^		23^+6^		33		38	
BMI, kg/m^2^	33.2		26.3		38		27.7	
DOS at admission, days	11		8		7		1	
DOS at HP, days	12		9		8		5	
SOFA	2		2		2		3	
APACHE II	3		16		3		3	
Hospital LOS, days	18		14		18		15	
ICU LOS, days	14		8		14		12	
IMV day, days	13		NA		NA		8	
Frequency of HP, number	3		3		2		3	

	Pre	Post	Pre	Post	Pre	Post	Pre	Post
CRP, mg/L	81.04	8.12	139.45	28.52	106.86	23.63	253.33	12.55
IL-6, pg/mL	13.45	1.97	6.11	2.12	9.34	5.06	N/A	N/A
Ferritin, ng/mL	513	N/A	1556	N/A	N/A	N/A	N/A	N/A
PCT, ng/mL	0.07	0.07	1.18	0.6	0.26	0.18	1.5	0.31
PaO_2_, mmHg	97.3	122	116	147	N/A	180	85	174
SpO_2_	95	99	100	99	100	99	98	99
PaO_2_/FiO_2_ ratio	195	244	290	368	363^a^	483^a^	121	348
Hb, g/dL	11.7	11.6	9.7	10.7	11.7	10.3	10.9	10.8
Lymphocyte count, /μL	1173	949	165	151	1006	689	771	1006
Platelet, /μL	180	211	237	210	191	240	170	145
Creatinine, mg/dL	0.3	0.32	2.58	1.55	0.82	0.59	0.63	0.72
Heart rate, bpm	96	126	60	87	104	88	51	60
MAP, mmHg	81	79	89	126	96	88	90	91
RR, bpm	30	22	22	18	30	20	26	14

Neonatal characteristics								
GA at delivery, weeks	40^+1^		37^+5^		38		38	
Birth weight, g	3520		2120		3200		2890	

APACHE II, acute physiology and chronic health evaluation II; BMI, body mass index; bpm, beats per minute or breaths per minute; CRP, C-reactive protein; DOS, day of symptom; GA, gestational age; Hb:, hemoglobin; HP, hemoperfusion; ICU, intensive care unit; IL-6, interleukin 6; IMV, invasive mechanical ventilation; LOS, length of stay; MAP, mean arterial pressure; PF, PaO_2_/FiO_2_ ratio; PCT, procalcitonin; pre, before hemoperfusion; post, after hemoperfusion; RR, respiratory rate; SOFA, sequential organ failure assessment score

^a^SpO_2_/FiO_2_ ratio

In this single-center case series, the patients' ages ranged from 21 to 36 years old, and their gestational ages at the time of admission ranged from 18 to 38 weeks. Mechanical ventilation was required for two patients (patient number 1 and 4), but they were successfully extubated. HP was performed approximately 7 days (ranging from 5 to 12 days) after the onset of symptoms (DOS), and all patients had CRP levels exceeding 80 mg/dL before treatment. Each patient received around three sessions of HP. Following treatment, there was an improvement in oxygenation (indicated by an increased PaO_2_/FiO_2_ ratio) and a decrease in CRP levels. In this series, none of the patients required renal replacement therapy or received vasopressors during their intensive care unit (ICU) admissions.

### Treatment protocol in COVID-19

All participants received standard critical care management for COVID-19 according to local guidelines adapted from World Health Organization (WHO) guidelines [17, 18]. Briefly, remdesivir was used as the first-line antiviral therapy and short courses of systemic corticosteroids were used in severe to critical illness, as classified by WHO. Immunomodulators were considered for patients with worsening hypoxemia and increased cytokine levels (i.e., interleukin-6 and CRP). Extracorporeal Blood purification with hemoperfusion technique was considered as the alternative treatment when contraindications for using immunomodulators were presented.

A 3 hour session of HP with a cytokine adsorptive cartridge (HA330^®^, Jafron, China) was performed for three to five consecutive days. The blood flow rate was set at 150–200 mL/minute. Prior to use, the cartridge was primed with 5000 units of unfractionated heparin for 30 minutes and then flushed with 2 L of 0.9% sodium chloride solution. A double lumen catheter was inserted into the right internal jugular vein.

### Obstetric and neonatal outcome

All newborns were delivered by cesarean section. The cesarean delivery was elective and performed due to non-COVID-19-related obstetric indications in one case (patient number 2), while emergency cesareans were performed in cases related to maternal obstetric indications, such as hemolysis, elevated liver enzymes, and low platelet count (HELLP) syndrome in patient number 1 (failed induction of labor) and spontaneous onset of labor in patients number 3 and 4. All neonates had a gestational age of higher than 37 weeks. There were no maternal or neonatal deaths or related complications reported in the present study.

### Complications

No HP-related complications, such as hemodynamic instability including hypotension and arrhythmia, catheter-related complications, local bleeding, thrombocytopenia, or hypocalcemia, were reported in this case cohort. In the observed cases of HELLP syndrome, there was no serious thrombocytopenia after HP.

## Discussion

We reported on four pregnant patients with severe COVID-19 disease who received extracorporeal blood purification using the HA330^®^ adsorptive cartridge. All patients showed favorable clinical outcomes, and there were no complications related to this treatment modality in this cohort.

Pregnant women infected with SARS-CoV-2 have an increased risk of severe illness and are independently associated with worse outcomes compared with nonpregnant women of similar ages [19]. Additionally, COVID-19 during pregnancy has been linked to an increased risk of delivering a preterm (earlier than 37 weeks) or stillborn infant [20]. The pregnant women with COVID-19 had a higher risk of ICU admission, the need for ventilatory support compared with nonpregnant women, and increased risk of maternal death [5, 21, 22]. Due to physiological changes in the respiratory system during pregnancy, the risk of respiratory compromise is increased [4]. Furthermore, Muthuka *et al*. reported that pregnant women were more susceptible to cytokine storms compared with nonpregnant women [23]. This relationship may be explained by elevated cytokine levels and the overexpression and upregulation of the angiotensin-converting enzyme 2 (ACE2) receptor during pregnancy [24, 25]. The ACE2 receptor is the primary entry site for SARS-CoV-2 resulting in a higher likelihood of COVID-19 disease [26]. Therefore, both immunomodulators and HP may have a potential role in treating pregnant patients with severe COVID-19. According to the National Institutes of Health (NIH) COVID-19 treatment guidelines, IL-6 inhibitors (sarilumab, tocilizumab) and Janus kinase (JAK) inhibitors (baricitinib, tofacitinib) are currently used in the treatment of severe COVID-19 cases that require supplemental oxygen, high-flow oxygen, noninvasive ventilation (NIV), mechanical ventilation, or extracorporeal membrane oxygenation (ECMO) [27]. However, the use of these immunomodulatory drugs in pregnant women carries potential risks. Tocilizumab has limited available data in pregnant women, with the Australian Therapeutic Goods Administration (TGA) classifying it as pregnancy category C due to potential risk to the fetus based on animal data [28]. Baricitinib has been associated with serious adverse events and embryofetal toxicities, including skeletal anomalies and reduced fertility in animal studies [29]. Therefore, the use of these immunomodulatory drugs may have detrimental effects on pregnant patients. In our institution, we opted to perform HP with the cytokine adsorber HA330^®^ (Jafron, China) as adjunctive therapy for immunomodulation instead of tocilizumab and baricitinib.

HP using Jafron HA series (such as HA330^®^ and HA380^®^) is theoretically effective in modulating severe inflammatory processes by nonselectively adsorbing and removing cytokines from the bloodstream [30]. Previous studies in patients with severe COVID-19 pneumonia have demonstrated that HP with the cytokine adsorber HA330^®^ was associated with significant removal of inflammatory factors (IL-6, CRP, and ferritin) from the circulation, decreased hospital mortality and length of hospitalization, improved oxygenation, and had no safety concerns [12–16]. Furthermore, the hemoadsorption technique can be terminated at any time without long-term immunosuppressive effects compared with immunomodulators. Nonetheless, there is limited data on using this treatment in pregnant women, who are typically not included in studies, and only case reports have been published [31, 32].

The findings of this study are consistent with the results of other studies evaluating the therapeutic effect of HP with cytokine adsorber in patients with COVID-19 [12–16]. Soleimani *et al*. reported significant increases in SpO_2_ and decreases in CRP levels in patients who underwent adjunctive HP with cytokine adsorber (HA380 or HA280 cartridge) compared with conventional treatment [14].

All pregnant women in our study delivered healthy babies without any documented perinatal complications. There were no serious adverse events related to the use of HP with cytokine adsorptive cartridge, but most of studies were either case series or had relatively small sample sizes [33, 34]. The potential treatment-related complication was bleeding due to reduction in platelet count [33, 34]. However, these implied that HP with cytokine adsorber is a feasible and relatively safe procedure in pregnancy. The findings of this study may help guide the selection of HP as an alternative treatment in pregnant patients when standard immunomodulatory therapy is contraindicated or unavailable during a pandemic period. Nonetheless, it is imperative to investigate the potential relationship between HELLP syndrome and complications associated with HP, particularly thrombocytopenia. Additional research is required to clarify this association.

We acknowledge several limitations of this study. First, due to the small sample size and single-center nature of the study, it is not possible to draw definitive conclusions about the exact outcomes of COVID-19 in maternal and neonatal health. However, our case series provides valuable information regarding efficacy and safety in pregnant patients. Secondly, while this study demonstrated the feasibility and safety of HP using HA 330^®^, the long-term maternal and infant outcomes remain unclear. Lastly, we did not evaluate other adverse effects of adsorptive techniques, such as the potential impact on therapeutic antibiotic blood levels, which could affect clinical outcomes. Future studies are needed to confirm or refute these findings.

## Conclusion

This small case series demonstrated the feasibility and relative safety of using HP with the HA330 cartridge as an adjunctive treatment during the cytokine storm phase in pregnant patients with severe COVID-19.

## Data Availability

All the data generated or analyzed during this study are included in this article. Further inquiries can be directed to the corresponding author.
